# Evolution of two distinct phylogenetic lineages of the emerging human pathogen *Mycobacterium ulcerans*

**DOI:** 10.1186/1471-2148-7-177

**Published:** 2007-09-27

**Authors:** Michael Käser, Simona Rondini, Martin Naegeli, Tim Stinear, Francoise Portaels, Ulrich Certa, Gerd Pluschke

**Affiliations:** 1 Swiss Tropical Institute, Socinstr. 57, 4002 Basel, Switzerland; 2 Department of Microbiology, Monash University, Wellington Rd, Clayton 3800, Australia; 3 Institute of Tropical Medicine, Antwerp 2000, Belgium; 4 F. Hoffmann-La Roche Ltd., 4070 Basel, Switzerland

## Abstract

**Background:**

Comparative genomics has greatly improved our understanding of the evolution of pathogenic mycobacteria such as *Mycobacterium tuberculosis*. Here we have used data from a genome microarray analysis to explore insertion-deletion (InDel) polymorphism among a diverse strain collection of *Mycobacterium ulcerans*, the causative agent of the devastating skin disease, Buruli ulcer. Detailed analysis of large sequence polymorphisms in twelve regions of difference (RDs), comprising irreversible genetic markers, enabled us to refine the phylogenetic succession within *M. ulcerans*, to define features of a hypothetical *M. ulcerans *most recent common ancestor and to confirm its origin from *Mycobacterium marinum*.

**Results:**

* M. ulcerans *has evolved into five InDel haplotypes that separate into two distinct lineages: (i) the "classical" lineage including the most pathogenic genotypes – those that come from Africa, Australia and South East Asia; and (ii) an "ancestral" *M. ulcerans *lineage comprising strains from Asia (China/Japan), South America and Mexico. The ancestral lineage is genetically closer to the progenitor *M. marinum *in both RD composition and DNA sequence identity, whereas the classical lineage has undergone major genomic rearrangements.

**Conclusion:**

Results of the InDel analysis are in complete accord with recent multi-locus sequence analysis and indicate that *M. ulcerans *has passed through at least two major evolutionary bottlenecks since divergence from *M. marinum*. The classical lineage shows more pronounced reductive evolution than the ancestral lineage, suggesting that there may be differences in the ecology between the two lineages. These findings improve the understanding of the adaptive evolution and virulence of *M. ulcerans *and pathogenic mycobacteria in general and will facilitate the development of new tools for improved diagnostics and molecular epidemiology.

## Background

*M. ulcerans *is the causative agent of the chronic necrotising human skin disease Buruli ulcer. After tuberculosis and leprosy, Buruli ulcer is the third most common mycobacterial disease, and Western Africa is the world region most affected. The disease usually begins as a painless nodule and, if left untreated, leads to massive tissue destruction. More than 50% of those affected by Buruli ulcer are children under 15 years of age. The disease often occurs in focalised areas close to stagnant or slow-moving waters. The mode of transmission is thought to be from environment to human but is still very poorly understood, partly because standard molecular typing methods lack the resolution required for detailed micro-epidemiological analyses.

Whole genome sequence comparisons of an *M. ulcerans *isolate from Ghana (Agy99) with the *M. marinum *M strain have shown that the former has evolved from the latter by a process of lateral gene transfer and reductive evolution [1,2]. Characteristic for *M. ulcerans *and probably a key driver of its speciation is the acquisition of the virulence plasmid, pMUM001, required for production of the tissue damaging polyketide, mycolactone [3,4]. Another striking feature of the *M. ulcerans *Agy99 genome was the many examples of DNA deletions when compared with the *M. marinum *M strain which were referred to as MURDs (*M. ulcerans *regions of difference, [5]) and account for the loss of 1000 kb of DNA between *M. marinum *and *M. ulcerans*.

For other mycobacterial pathogens such as *Mycobacterium tuberculosis*, *M. leprae*, and *M. avium*, inter- and intra-species comparative genomics has contributed considerably to our understanding of their evolution, virulence and phylogeographical dispersal [6-16]. Especially, specific deletions in regions of difference (RDs) proved to be excellent epidemiological and evolutionary markers since they did not occur independently in different strains but rather result from events in a common progenitor [8]. Thus, to gain further insight into *M. ulcerans *and explore the DNA deletion diversity among *M. ulcerans *strains we recently developed a plasmid-based DNA microarray that facilitated the detection of large sequence polymorphisms among *M. ulcerans *isolates of world-wide origin [17]. These initial microarray studies revealed twelve deletions (in twelve regions of difference, designated RD1 to RD12) between 2 and 53 kb in size among the 30 *M. ulcerans *isolates tested, representing hitherto unknown large sequence polymorphisms and uncovering a major source of strain diversity in *M. ulcerans*, a species where nucleotide diversity is less than 0.6% even between the most distantly related strains [2]. This insertional-deletional (InDel) genomic variation showed that genome reduction is ongoing within *M. ulcerans *which provides evidence for an adaptive change from an environmental to a possibly new host-adapted organism.

In this current study, we have undertaken a detailed characterization of these twelve RDs comprising over 410 kb based on InDel events that allowed for a phylogenetic resolution, of a representative collection of 35 *M. ulcerans *patient isolates of world-wide origin for which genotyping was very limited. Most importantly, we show the existence of two distinct phylogenetic lineages with diverse evolutionary history in *M. ulcerans *which has implications for both the understanding of mycobacterial adaptation and further research on this emerging human pathogen.

## Results

### Identification and localisation of genomic regions of difference (RDs) in *M. ulcerans*

**Figure 1:**
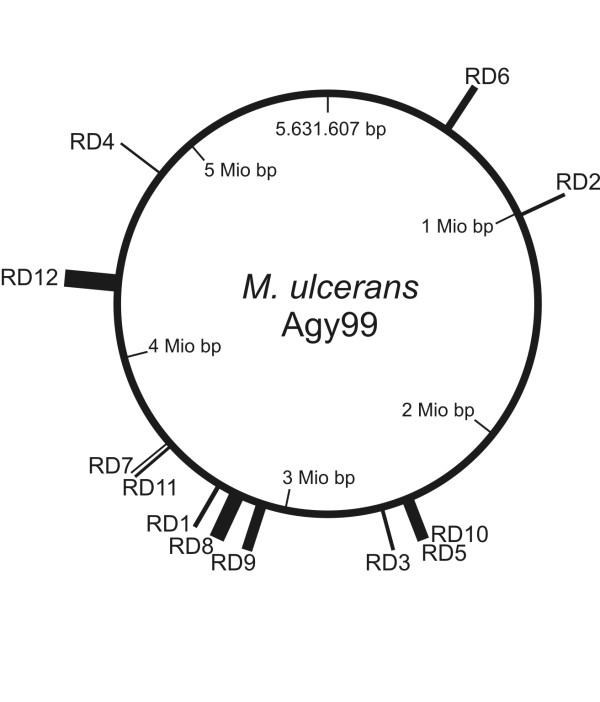
**Positions of RD1 to RD12 on the *M. ulcerans *genome Agy99**. Widths of the bars correspond to the sizes of deletions.

**Figure 2:**
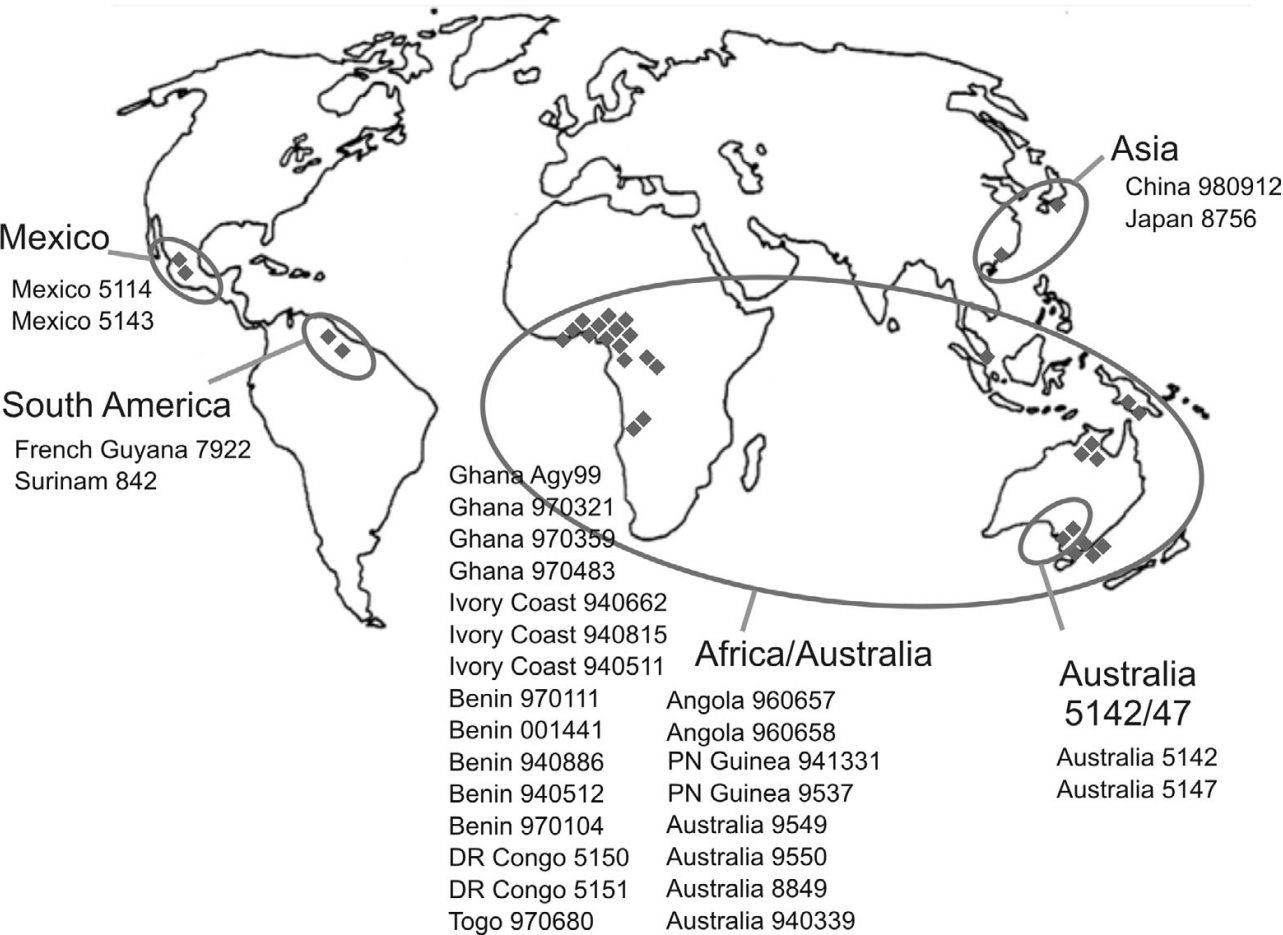
**Geographical distribution of the five *M. ulcerans *haplotypes**. The origin of *M. ulcerans *strains included in this study is shown in the world map, with each dot representing one patient isolate as defined in materials and methods. The five InDel haplotypes are encircled.

In a previous study we identified twelve RDs among 30 *M. ulcerans *strains of diverse geographic origin using a DNA microarray based on the Ghanaian reference strain Agy99 [17]. For the current investigation, we mapped each RD on the recently completed Agy99 genome (Fig. 1). Five of the RDs were located on the genome between 3.0 and 3.6 Mbp. The other seven identified RDs were distributed elsewhere on the chromosome. As found upon in depth analysis (see below), the twelve RDs altogether spanned some 410 kb, representing more than 7% of the *M. ulcerans *Agy99 genome (Fig. 1). Size analysis of the deletions clustered the 30 analysed *M. ulcerans *strains of diverse geographic origin into five haplotypes (where haplotype is defined as a set of DNA polymorphisms inherited as a unit). The geography of the haplotypes and the origins of the *M. ulcerans *strains under investigation are shown in a distribution map (Fig. 2).

### Complete analysis of large sequence polymorphisms in *M. ulcerans *RDs confirms five haplotypes

To further resolve the above microarray based phylogenetic differentiation we analysed each of the twelve RDs in greater detail by focussing on two independent patient isolates for each of the five haplotypes. Since the method used for detection of deletional diversity [17] would bias the results towards phylogenetically informative events leading away from the reference strain Agy99, we monitored the genome composition of the RDs irrespective of the information gained by the microarray approach and referred to the *M. marinum *M strain sequence. Using PCR, cloning and primer walking we determined deletion sizes and their breakpoints, and identified sequence insertions, substitutions, dislocations, inversions and rearrangements. For crucial loci, confirmatory tests were made for the whole and extended collection of 35 *M. ulcerans *strains. Consistently throughout our analysis, members of a given subgroup yielded identical results (see below) in all RDs analysed and confirmed the occurrence of five haplotypes. Thus, strain Japan 8756 was identical to China 98912 as was Surinam 842 to French Guyana 7922 and the two Mexican isolates 5114 and 5143 to each other, defining haplotypes referred to as the Asian, the South American and the Mexican, respectively. The Asian haplotype excludes strains of South East Asian origin. Comparative analysis of the largest subgroup of strains, comprising the isolates originating from Africa, Australia, Papua New Guinea and Malaysia, revealed no large sequence polymorphisms within the subgroup and represented the African/Australian haplotype. Two of the Australian strains, 5142 and 5147, are almost identical to the African/Australian haplotype but have an additional deletion and thus represent a separate haplotype, Australia 5142/47. Since identical results were obtained for all independent isolates per haplotype we conclude that the large sequence polymorphisms identified were neither experimental artefacts nor events that had occurred during *in vitro *culturing over time. In contrast, these concordant InDels reflect real geographically associated features with the genome rearrangements resulting from irreversible genetic events that had occurred in the common progenitor strains of each haplotype. Thus, we consider the description of InDels as useful phylogenetic markers since *M. ulcerans *strains appeared to be largely clonal [18-21] and recombination is unlikely to occur extensively in this species [22].

### Detailed RD sequence comparison reveals the existence of two major *M. ulcerans *lineages

Genome sequence polymorphism data were compared with the available reference complete genome sequences of *M. ulcerans *Agy99 (a member of the African/Australian haplotype) and the *M. marinum *strain M. Properties of the five *M. ulcerans *haplotypes are presented in Table 1 in comparison to the *M. marinum *M sequence. In the genomes of the South American, Mexican and Asian haplotypes deletions in the absences of substituting DNA such as an insertion sequence element (ISE) are more frequent and the deletions are larger than in the African/Australian cluster (Table 1, column 1). In contrast, insertions of ISEs (IS*2404*, IS*2606*, and IS*2404*/IS*2606 *tandems, Table 1, column 2) were frequently found in the African/Australian haplotypes, but not in the South American, Mexican and Asian haplotypes. Moreover, in the African/Australian cluster a multitude of genomic rearrangements was observed, including i) large DNA fragment dislocation from remote sequence positions in the *M. marinum *genome into the investigated RDs (Table 1, column 4); ii) DNA fragment inversions (Table 1, column 5); and iii) DNA fragment rearrangements involving sequences derived from unlinked *M. marinum *loci that are rearranged and then linked to each other by IS*2404 *elements (Table 1, column 6). Such a rearrangement was not found in any of the twelve RDs for the South American, Mexican and Asian haplotypes. These *M. ulcerans *haplotypes thus shared a genetic backbone corresponding to the *M. marinum *strain M sequence at loci where the African/Australian haplotype (including the *M. ulcerans *genome reference strain Agy99) showed extensive genome rearrangements. DNA sequences present in the South American, Mexican and Asian haplotypes and missing in the African/Australian haplotypes showed an overall sequence identity of 98% with the corresponding sequences in the *M. marinum *strain M.

**Table 1:** Genomic properties of *M. ulcerans *haplotypes as compared to the *M. marinum *strain M sequence in the twelve RDs

	**deletions only**		**insertions only ^b^**		**deletion::insertion ^b^**		**fragments dislocated**		**fragments inverted**		**fragments rearranged involving IS*2404***	
	size (kb)	in RD	quantity (qualifier)	in RD	size (kb)	in RD	quantity	in RD	quantity	in RD	quantity	in RD
	5.0	RD2										
	0.8 ^a^	RD3A										
					11.1::IS*2404 *^c^	RD1						
	1.8	RD4										
***M. ulcerans *South America**					3.8::IS*2404*	RD3B						
	7.3	RD7					1	RD12	1	RD12		
					17.2::14.7 ^d^	RD7						
	0.5	RD10										
					30.5::IS*2404*	RD9B						
	3.9 ^a^	RD12A										
	27.5	RD12B										

	0.8 ^a^	RD3A										
	60.7	RD5,10										
***M. ulcerans *Mexico**					4.6::IS*2404*	RD11A						
	51.5	RD8										
	3.9 ^a^	RD12A										

	0.8 ^a^	RD3A										
	24.1	RD9A										
	3.6	RD9										
***M. ulcerans *Asia**					4.6::IS*2404*	RD11A						
	3.4	RD10										
	3.9 ^a^	RD12A										
	42.0	RD12C										

			3 (IS*2404*)	RD1								
			1 (IS*2404*)	RD3								
			1 (IS*2404*)	RD10								
			2 (IS*2404*)	RD5								
			2 (IS*2404*)	RD6								
			1 (IS*2404*) ^c^	RD7	1.7::IS*2404/2606*	RD1						
							2	RD2	1	RD2		
	0.8 ^a^	RD3A	1 (IS*2404*)	RD8							1	RD2
							3	RD5,10	2	RD5,10		
***M. ulcerans *Africa/Australia**	2.5	RD8A	3 (IS*2404*)	RD12	2.1::0.3 ^d^	RD2					3	RD5,10
							1	RD6	2	RD6		
	1.8	RD9C	2 (IS*2606*)	RD2	1.0::15.7 ^d^	RD6					1	RD6
							2	RD8	1	RD8		
	3.9 ^a^	RD12A	1 (IS*2606*)	RD10	6.9::IS*2404*	RD8					2	RD8
							1	RD9	1	RD9		
			5 (IS*2606*)	RD7	4.2::IS*2404*	RD9D						
			1 (IS*2606*)	RD8								
			1 (IS*2606*)	RD11								
			1 (IS*2404*/*2606*)	RD2								
			1 (IS*2404*/*2606*)	RD6								
			2 (IS*2404*/*2606*)	RD12								

***M. ulcerans *Australia 5142/47**	as above, additionally: 3.5 (RD3C)		as above		as above		as above		as above		as above	

a: these two deletions are common for all investigated *M. ulcerans *strains

b: inserts consist of complete IS elements plus some additional flanking nucleotides

c: this insertion consists of one functional IS*2404 *element and an additional truncated IS*2404 *fragment

d: the inserted DNA stretches are not represented in the *M. marinum *strain M genome sequence; gene bank blast and fasta searches identified sequences of i) bacteria other than mycobacteria with about 60% nucleotide identity over up to 3100 bp for the InDel in RD7; ii) mammalia with about 68% identity over 320 bp for the InDel in RD2; and iii) mycobacteria and other environmental or pathogenic bacteria with identities between 57 and 83% over 600–1900 bp, most of them grouping into insertion sequences and phages, for the InDel in RD6.

**Figure 3:**
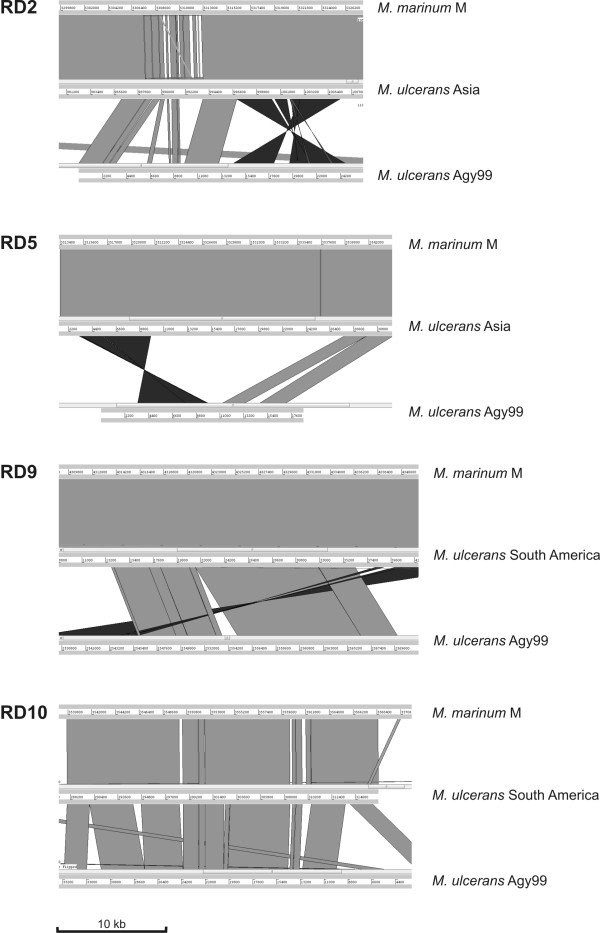
**Linear genomic comparison of sections within RDs**. Comparisons are made with three sequences each using ACT (the Artemis Comparison Tool software release 5) over at least 30 kb: *M. marinum *M on top, *M. ulcerans *Agy99 at the bottom, and *M. ulcerans *of either haplotype, the Asian (RD2 and RD5) or the South American (RD9 and RD10) in the middle. Regions of sequence conformity are shown in parallel light grey plains, inverted DNA segments are depicted in dark grey and inverted surfaces, and white areas represent non-homologous regions like deletions and insertions. Some sequence displacements are visualized as grey areas displaying across the panels. Cut-off value for inclusion of sequence identity was 100 bp. The principal genetic backbone of the Asian and South American haplotypes (both members of the ancestral lineage) is identical for each alignment shown, but – as a matter of how the RDs were found – the particular excluded haplotypes reveal deletions in the respective RDs. Although showing the same genetic backbone as *M. marinum *in the marginal parts, the Mexican strains disclose large deletions over their respective RDs and are therefore not included in this computational analysis. The sequence regions were retrieved by scanning the contigs by PCR, and by cloning and sequencing of critical segments.

The twelve RDs thus distinguish two major *M. ulcerans *lineages: one branch, comprising the isolates from Africa, Australia, Malaysia and Papua New Guinea, we have called the *classical lineage*, since it includes the sequenced African strain, Agy99, and most of the existing *M. ulcerans *clinical isolates. The second lineage comprises the strains of Asian, South American and Mexican origin. We designated it the *ancestral lineage*, since its members are genetically closer to the progenitor *M. marinum *in sequence composition, order and orientation. This is illustrated for selected RDs in Fig. 3 where the sequence of *M. marinum *is aligned to each one representative haplotype of the *M. ulcerans *ancestral lineage and to *M. ulcerans *Agy99, representing the classical lineage. The alignments demonstrate the high conformity between *M. marinum *and members of the ancestral *M. ulcerans *lineage with only minor changes including single nucleotide polymorphisms, small deletions or sequence variations over short stretches. In contrast, major genome rearrangements mark significant genomic differences between the ancestral and the classical lineage (Fig. 3).

### Irreversible sequence polymorphisms disclose phylogenetic relationships and an evolutionary scenario for *M. ulcerans*

**Figure 4:**
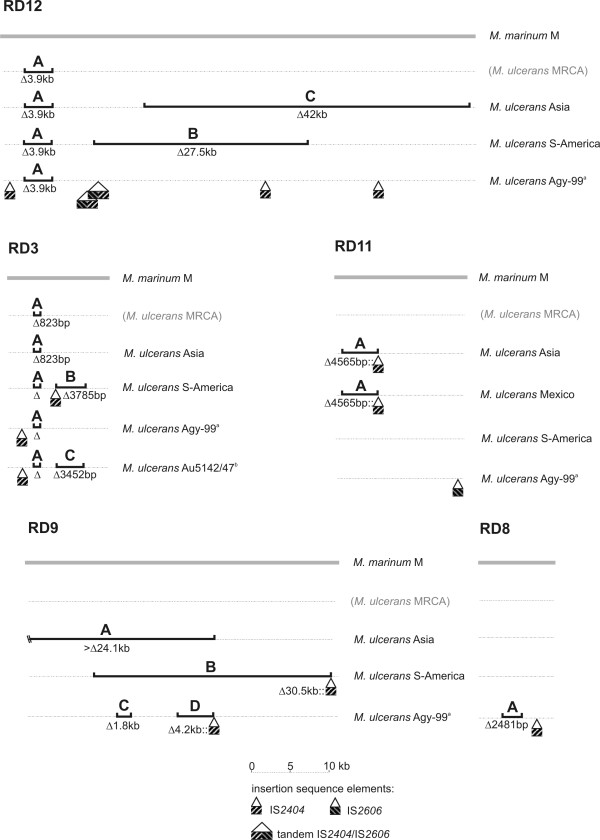
**Genome comparison of *M. marinum *strain M and *M. ulcerans *haplotypes in selected RDs**. Only selected RDs that contribute to the understanding of the *M. ulcerans *phylogeny are shown. Dashed lines represent sequence identity; Δ = deletion; :: = substitution; shaded boxes are IS*2404 *and IS*2606 *as indicated. *M. ulcerans *MRCA = most recent common ancestor. Since the Mexican strains showed either deletions expanding the whole RD or did not show any significant microarray hit in other RDs, the Mexican haplotype was only included in the illustration of RD11 where it revealed informative differences. a) Alignment of the members of the African/Australian haplotype shows sequence identity in the tested crucial genome regions. b) Haplotype Australia 5142/47 is identical in all regions except for RD3C as indicated; here the breakpoints differ from deletion RD3B in the South American haplotype.

The two deletions RD12A (the 3.9 kb deletion in RD12) and RD3A (the 0.8 kb deletion in RD3; Table 1) were shared by all *M. ulcerans *strains analysed. These shared features define the hypothetical *M. ulcerans *most recent common ancestor (MRCA) from which the two major lineages descended. Acquisition of the virulence plasmid, pMUM001, is also a characteristic of the MRCA. In Fig. 4, haplotype specific configurations of insertional-deletional polymorphisms are shown for five selected RDs. The deletional patterns within a given RD differ across the haplotypes and the deletions within one RD were given letter extensions (A-D, Fig. 4 and Table 1). Sequence position details of these deletions are summarized in Table 2. The configurations within several loci provide a non-ambiguous picture of the phylogenetic relationship between the five *M. ulcerans *haplotypes. In Fig. 4, comparative analysis of RD12 shows that the Asian, South American and African haplotypes share the 3.9 kb deletion, a feature of the *M. ulcerans *MRCA. Apart from this, none of the three subgroups can have descended from each other, since each of them has either maintained DNA stretches of the *M. marinum *genetic backbone that are deleted in the other genotypes (RD12B for the South American and RD12C for the Asian haplotype) or has accumulated insertions that are missing in the others (ISEs IS*2404 *and IS*2606 *in RD12 for Agy99, Fig. 4). Sequence comparison in RD8 illustrates that neither the Asian nor the South American strains can have derived from the African strain Agy99 due to the absence of both the African-Australian specific deletion RD11A and IS*2404 *insertion (Fig. 4). In contrast, alignments in RD9 show that Agy99 cannot have one of the ancestral haplotypes as an ancestor since it has maintained stretches that were deleted in either of them. Similar conclusions can be drawn from sequence comparison in RD3 which also shows the derivation of the two strains Australia 5142 and 5147 from the African/Australian cluster (Fig. 4). Interestingly, in RD3 both the South American and the Australian haplotype of strains 5142 and 5147 carry a deletion at the same position, but with different sizes (3785 bp of RD3B versus 3452 bp of RD3C) and different breakpoints at each of their flanking sequences. Furthermore, an IS*2404 *element has been inserted in the South American haplotype, while no substituting insertion is found in the Australian strains indicating that the two deletions have evolved by different mechanisms (Fig. 4). Partly overlapping deletions that also appear to have arisen independently have also been found in a number of other RDs (e.g. RD9 and 12) suggesting that some loci are hot spots for genomic changes.

**Table 2:** Positions of deletions used for the phylogenetic description

**Deletion**	**Size (bp)**	**Position in *M. marinum *sequence**	**corresponds to**
RD3A	823	3.702.623 – 3.703.446	
RD3B	3785	3.705.487 – 3.709.281	
RD3C	3452	3.705.557 – 3.709.018	
RD8A	2481	1.395.048 – 1.398.043	MURD25
RD9A	> 24067	4.338.150 – 4.362.217	
RD9B	30474	4.348.127 – 4.378.601	
RD9C	1763	4.348.699 – 4.350.461	MURD94
RD9D	4230	4.357.791 – 4.362.021	MURD95
RD11A	4565	3.108.140 – 3.112.725	
RD12A	3938	4.899.809 – 4.903.746	MURD105
RD12B	27484	4.908.774 – 4.936.258	
RD12C	41961	4.915.409 – 4.957.370	

Positions refer to the *M. marinum *M genome sequence [34] following Stinear et al., 2007 [5]. Discrepancies between position numbers and deletion sizes are due to nucleotide variations between the *M. marinum *and *M. ulcerans *genomes in these regions

**Figure 5:**
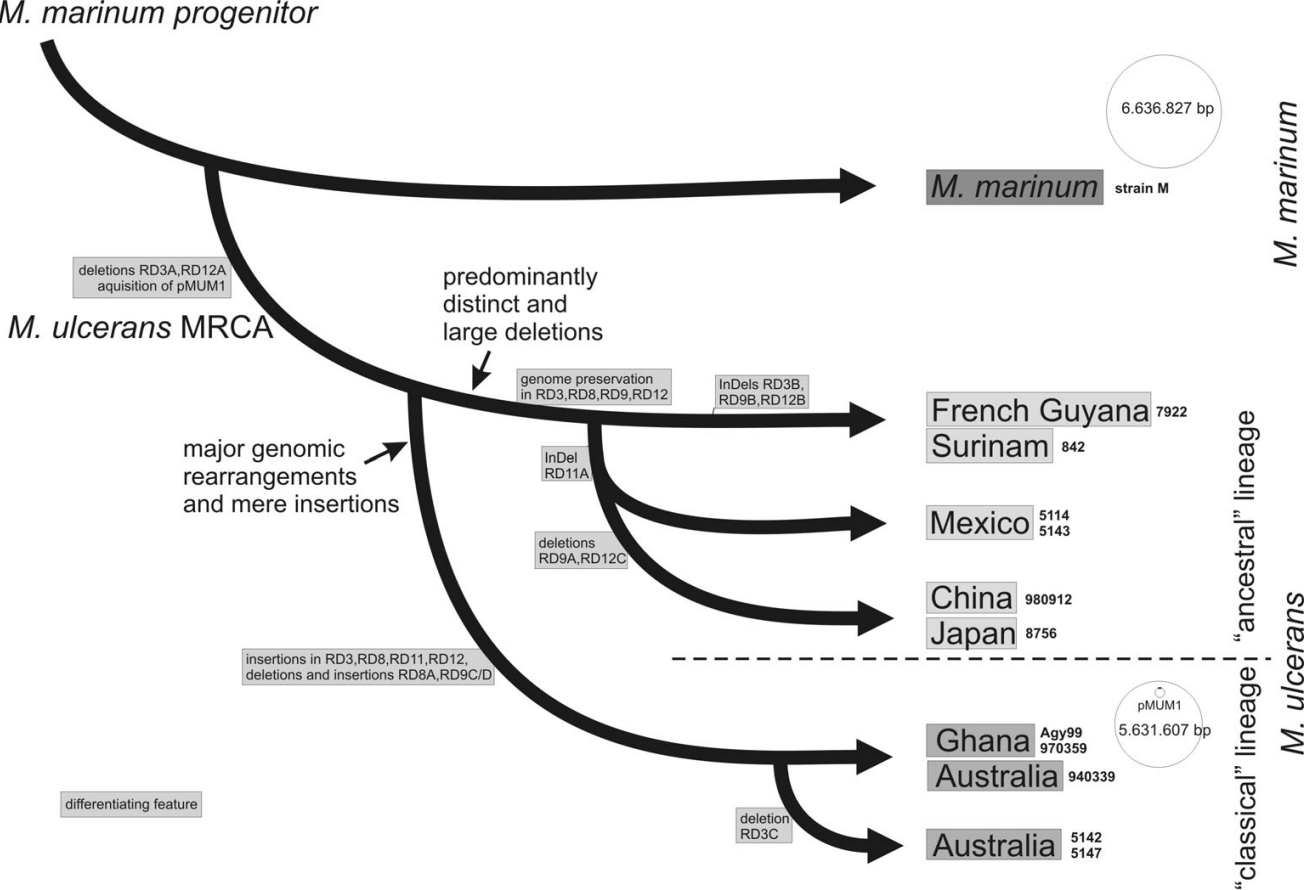
**Evolutionary scenario for *M. ulcerans*, basically distinguishing two major lineages, according to the RDs analyzed in this study**. All strains with a strain identifier added to the right depict recent isolates. Note that both the *M. marinum *progenitor and the *M. ulcerans *MRCA are hypothetical strains. Features differentiating clusters or strains are dedicated to the branches between the nodes. RDs indicated here are all differentiated by features that are also shown in Fig. 4, whereas more RDs bear supporting features between the nodes (Table 1). The lengths of the internodes do not reflect time or genetic distance.

Other typing methods applied earlier to *M. ulcerans *isolates (IS*2404*-Mtb2 PCR, MIRU-VNTR and VNTR) resulted in dendrograms that equally position strains from Mexico, South America and (in two cases) also from Asia, members of the ancestral lineage, genetically closer to *M. marinum *than to the cluster of African, Australian and South East Asian isolates, members of our classical lineage [23-25]. Two recent studies based on MLST also placed the branching point of a Surinam, Mexican and a Chinese isolate at the junction between a cluster of each one African, Australian and South East Asian *M. ulcerans *strain and various *M. marinum *types [1,2]. Here, albeit with yet low geographical resolution, an unequivocal evolutionary scenario can be proposed for *M. ulcerans *haplotypes, in which all branching points are well defined by irreversible and non-ambiguous genetic markers (Fig. 5). The *M. ulcerans *MRCA (and with it all recent *M. ulcerans *isolates) is distinguished from *M. marinum *strain M by the common deletions RD3A and RD12A. The classical lineage is separated from members of the ancestral lineage by numerous large sequence polymorphisms (Table 1) including at least seven genomic rearrangements in five RDs (RD2, 5, 6, 8, 10), blank insertions of ISEs in ten RDs (all twelve except RD4 and 9) and deletions in seven RDs (RD1, 2, 3, 6, 8, 9, 12; Table 1). Within the classical lineage, the Australian strains 5142 and 5147 separate by RD3C (Fig. 4 and 5) from all other members of this lineage. The three haplotypes belonging to the ancestral lineage are separated from each other by deletions of considerable size such as the partially overlapping but independent deletions in RD12 (RD12C of 42 kb and RD12B of 27.5 kb) and in RD9 (RD9A of > 24 kb and RD9B of 30.5 kb) in the Asian and South American haplotypes, respectively (Fig. 4 and 5). Interestingly, a shared InDel event in RD11 (RD11A of 4565 bp substituted by an IS*2404 *element, Fig. 4 and 5) suggests a closer relationship between the Mexican and Asian than between the Mexican and South American haplotypes.

## Discussion

Large genome sequence polymorphisms have been used to unravel inter-species relatedness and evolutionary order within the *M. tuberculosis *complex as well as for other mycobacterial species [8,22,26]. Our microarray based comparative genomic hybridization analysis of *M. ulcerans *isolates demonstrates that InDel diversity is also common in this mycobacterial species [17]. A detailed analysis of the twelve identified RDs presented here defined properties of a hypothetical *M. ulcerans *MRCA, and identified two major distinct lineages among *M. ulcerans *strains, which could all be assigned to either a classical or an ancestral lineage. Members of the ancestral lineage have a genomic backbone highly homologous to *M. marinum *and are therefore positioned closer to the *M. ulcerans *MRCA. Within the Asian, South American and Mexican haplotypes, a set of large, discrete and independent deletions could be identified upon comparison with the *M. marinum *strain M genome, while in members of the ancestral lineage no major genome rearrangements were found within the 410 kb of the investigated RDs (with one exception that showed no ISE involvement). In contrast, such changes were frequent in the isolates belonging to the classical lineage, where rearrangements of DNA fragments, at least partly caused by the activity of insertion sequence elements, led to complex genome reorganizations and interspersing of regions with other DNA fragments.

In our earlier microarray based analysis we hybridized genomic DNA from a set of *M. ulcerans *isolates belonging to the classical lineage to a panel of genomic fragments prepared from the sequenced reference strain Agy99 [17]. Although this approach favoured detection of InDel diversity within the classical lineage, only two subgroups could be distinguished within this lineage. While a single deletion of 3.45 kb in RD3C distinguished two Australian isolates from all other isolates belonging to the classical lineage, no additional differences were obtained with the 16 African, seven Australian, one Malaysian and two Papua New Guinean lineage members analysed. The prototype microarray used covered only 10% of the genome of strain Agy99 [17] and a whole genome array would be likely to identify more InDel diversity within the classical lineage.

The presence of irreversible genomic changes enabled us to unambiguously resolve an intra-species evolutionary scenario for *M. ulcerans*. The approach of InDel based phylogenetic analysis is independent of implied probabilities and has the advantage of giving a precise understanding of the direction of evolution of *M. ulcerans *strains. This evolutionary scheme advances the present descent information and is compatible with phylogenetic trees that have been proposed based on data obtained with other typing methods [23-25]. A recent report described several novel mycolactone-producing mycobacteria that were not associated with causing Buruli ulcer in humans [27], and subsequent MLSA suggested that they show very high affinity to *M. ulcerans *strains from South America [2,27]. We envision that application of the deletion analysis described here has the power to confirm and refine the phylogenetic relationship of these strains, where one would predict they belong to the *M. ulcerans *ancestral lineage.

All typing methods applied so far to *M. ulcerans *isolates from Africa and Australia revealed surprisingly few differences [18-21]. *M. tuberculosis *may have adapted to its human host far back in the beginning of human evolution [8,13], and *M. leprae*, the paradigm microbe for genome reduction, is so adapted to an intracellular lifestyle in human hosts that it is unable to grow in culture [28-30]. In comparison, *M. ulcerans *is suspected to have evolved more recently from an environmental bacillus to a mammalian pathogen [5,17]. Environmental changes, perhaps due to human activity, are suspected as a driving force for its emergence [31]. The diffuse picture of transmission possibilities of Buruli ulcer may reflect infection pathways that are more random than specifically evolved and human-adapted. The observed genome shrinkage of roughly 1 Mb from *M. marinum *to the classical lineage of *M. ulcerans *[1,5] probably reflects adaptation to a more stable environment(s) [17]. Preliminary inspection of the RDs showed that, apart from ISEs and phages, proteins involved in intermediary metabolism and respiration were prominent among the lost coding sequences (CDS) in all five *M. ulcerans *subgroups. Only in the Mexican haplotype a trend towards overproportional loss of proteins classified for virulence, detoxification, and adaptation was observed. In particular, in the classical lineage members of the PE/PPE gene families were highly represented in the repertoire of disrupted CDSs. Interestingly, four particular members of these protein families are eliminated in three of the five haplotypes by independent disruption processes. The fact that most cases of Buruli ulcer are caused by strains belonging to the classical lineage may either be indicative of a higher virulence in comparison to the ancestral lineage or of a higher prevalence in habitats relevant for transmission. It has to be further investigated whether severe Buruli ulcer lesions in the countries affected by *M. ulcerans *ancestral lineage strains are only occasional, as apparent from the clinical reports, or if additional cases presently remain either misdiagnosed or underreported. However, preliminary observations based on IS*2404 *identification in the Amazon region of Peru revealed low prevalence of Buruli ulcer disease although detection of IS*2404 *in the environment was similar to what was found in Benin (H. Guerra et al., submitted). Thus, it seems more likely that, after formation of the *M. ulcerans *MRCA from a *M. marinum *progenitor, the distinct genomic changes forged the classical lineage and rendered this emerging lineage more virulent. It is tempting to speculate that members of the ancestral lineage remained largely environmental mycobacteria that only occasionally affect humans in the endemic regions. The classical lineage haplotypes instead became widely dispersed, resulting in a clonal population within Africa and Australia. The ability to chronically infect mammalians, leading to shedding into the environment, may represent a property that is gaining importance for the survival of the species in highly endemic areas. A comprehensive comparison of the proteomes of the two *M. ulcerans *lineages may give insight into the differences of their adaptive biology.

## Conclusion

In this work, we present a detailed analysis of deletions, insertions, InDels, and genomic rearrangements by comparative genomics that distinguishes between five haplotypes of *M. ulcerans*, for which high-resolution genomic fingerprinting is still lacking. From this analysis, we have reconstructed the phylogenetic evolution of *M. ulcerans *in two distinct lineages, with the ancestral lineage being genetically closer to the environmental *Mycobacterium marinum*, and the classical lineage having undergone extensive genome reorganization and reduction. These findings contribute to the understanding of differences in pathogenicity across *M. ulcerans *isolates and sheds new light on the phylogeography of this emerging human pathogen. Distinction of subgroups within these *M. ulcerans *lineages leads us to conclude that InDels serve as evolutionary landmarks for differentiation within the species and help in the development of a genotyping strategy for both *M. ulcerans *and other environmental and pathogenic mycobacteria.

## Methods

### Mycobacterial strains and genomic DNA extraction

*M. ulcerans *clinical isolates used in this study are representative for the distribution and occurrence of cases and were as follows (further description of their origin is to be found in [23]): Ghana Agy99, Ghana ITM 970321, Ghana ITM 970359, Ghana ITM 970483, Ivory Coast ITM 940662, Ivory Coast ITM 940815, Ivory Coast ITM 940511, Benin ITM 970111, Benin ITM 940886, Benin ITM 940512, Benin ITM 970104, Democratic Republic of Congo (DRC) ITM 5150, DRC ITM 5151, Togo ITM 970680, Angola ITM 960657, Angola ITM 960658, Papua New Guinea ITM 941331, Papua New Guinea ITM 9537, Malaysia ITM 941328, Australia ITM 941324, Australia ITM 941325, Australia ITM 941327, Australia ITM 9549, Australia ITM 9550, Australia ITM 8849, Australia ITM 940339, Australia ITM 5142, Australia ITM 5147, China ITM 980912, Japan ITM 8756, French Guyana ITM 7922, Surinam ITM 842, Mexico ITM 5114, Mexico ITM 5143. Bacterial pellets of about 60 mg (wet weight) were heat inactivated for 1 hour at 95°C in 500 μl extraction buffer (50 mM Tris-HCl, 25 mM EDTA, 5% monosodium glutamate), and sequentially treated with lysozyme (2 h, 37°C, 17 M lysozyme) and proteinase K (overnight, 45°C, 0,3 M proteinase K in proteinase K buffer: 1 mM Tris-HCl, 5 mM EDTA, 0,05% SDS, pH7.8). After digestion, the samples were subjected to bead beater treatment (7 min, 3000 rpm, Mikro-Dismembrator S, B. Braun Biotech International, Melsungen, Germany) with 300 μl of 0.1 mm zirconia beads (BioSpec Products, Bartlesville, OK, USA). DNA was extracted from the supernatants by phenol-chloroform (Fluka, Buchs, Switzerland) extraction and subjected to ethanol precipitation. DNA concentration was measured by optical density at 260 nm (GeneQuant spectrophotometer).

### DNA methods

PCR was performed using FirePol 10× buffer and 0,5 μl FirePolTaq-Polymerase (Solis BioDyne, Tartu, Estonia), 2,5 ng genomic DNA, 0,6 μM forward and reverse primers each, 1,5 mM MgCl_2 _and 0,4 mM of each dNTP in a total volume of 25 μl. Long-range PCR polymerase mix (Fermentas, St. Leon-Rot, Germany) was applied according to the manufacturer's protocol to retrieve PCR products longer than 3 kb and up to 8 kb. PCR reactions were run in a GeneAmp PCR System 9700 PCR machine. The thermal profile for PCR amplification of *M. ulcerans *genomic DNA included an initial denaturation step of 95°C for 5 min, followed by 32 cycles of 95°C for 30 sec, annealing at 57°C for 30 sec, and elongation at 72°C for 30 sec to 4 min. The PCR reaction was finalized by an extension step at 72°C for 10 min followed by the analysis of the PCR products on 1–2% agarose gels by gel electrophoresis using ethidium bromide staining and the AlphaImager illuminator and AlphaImager software (Alpha Innotech, San Leandro, CA, USA). Primers were designed using the Primer3 software [32].

PCRs fragments produced for analysis of unknown genomic sequences were either purified using PEG800 precipitation and subjected to direct sequencing or cloned using the pGEM-T cloning kit (Promega, Wallisellen, Switzerland), transformed into JM109 (Sigma Aldrich, Buchs, Switzerland) bacterial cells, and sequenced after DNA preparation (Miniprep-Kit, Sigma Aldrich, Buchs, Switzerland). Sequencing was performed using the Big Dye kit and the AbiPrism310 genetic sequence analyzer (Perkin-Elmer, Waltham, MA, USA). Sequences were subjected to alignment and comparison with the AbiPrism Autoassembler version 1.4.0 (Perkin-Elmer, Waltham, MA, USA).

### Phylogenetic construction and DNA sequence analysis of RDs

Detailed phylogenetic reconstruction of the *M. ulcerans *collection was based on the detection of phylogenetically informative mutations over more than 410 kb including insertional-deletional diversity and genomic rearrangements as described in the following. Comparative genetic analysis of the RDs was achieved using a combination of PCR with perfect and/or degenerate primers, cloning, sequencing and primer walking. The *M. ulcerans *strain Agy99 genome sequence [33] and, in some instances, the *M. marinum *strain M (ATCC BAA-535) genome sequence were used as a template for PCR primer design [34]. For the five InDel haplotypes, we chose two strains each for PCR scanning and sequencing: Ghana 970359 and Australia 940339; Australia 5142 and Australia 5147; China 98912 and Japan 8756; French Guyana 7922 and Surinam 842; Mexico 5114 and Mexico 5143. Sequences of those strains were systematically aligned to the *M. marinum *M genome to identify and characterize InDels and genomic rearrangements. For each of these selected strains, between 1 and 3 kb of DNA was sequenced on each edge of the deletion. Insertions substituting the deletions were sequenced in total, and aligned genomic regions of the selected strains were scanned for their presence and size at least every 1 kb. All insertion elements within the 12 RDs were spanned using PCR in order to monitor their presence in the investigated strains. For crucial regions differing between the haplotypes, the whole strain collection was monitored by PCR. The resulting sequence information was subjected to comparative *in silico *sequence analysis including the *M. marinum *M strain and the *M. ulcerans *Agy99 strain sequence information.

### Data analyses and bioinformatics

Retrieved sequences were compared to the BuruList [35] and the *M. marinum *[36] blast servers and analysed using the sequence manipulation suite [37], the sequence alignment tool blast 2 sequences [38], and the Artemis software release 6 [39]. Some sequences were aligned to the *M. tuberculosis *H37Rv genome [40]. Linear genomic comparison was performed using the Artemis Comparison Tool software release 5 [41], with a cutoff value of 100 bp.

## Abbreviations

RD – regions of difference (including a sequence locus in which several genomic events may have led to various configurations)

InDel – Insertion-deletion (an event that includes an insertion substituting a deleted sequence in contrast to an insertion or a deletion only)

ISE – insertion sequence element (for *M. ulcerans*, two transposable elements are known as: IS*2404 *and IS*2606*)

## Authors' contributions

MK designed the molecular genetic studies, carried out the sequence alignments, developed the phylogenetic analysis and drafted and finalized the manuscript. SR carried out the microarray hybridizations and contributed to data acquisition. MN carried out the molecular genetic experiments. TS provided the sequence information and participated in approving the final manuscript. FP provided patient isolates and helped to finalize the manuscript. UC provided the microarray experimental facility and revised the manuscript critically for the content of evolutionary conclusions. GP supervised the project, participated in the design of the study and interpretation of the data and in the finalizing of the manuscript. All authors read and approved the final manuscript.
